# Assessment of Reproductive Endocrinology and Infertility Fellowship Programs Website

**DOI:** 10.7759/cureus.75754

**Published:** 2024-12-15

**Authors:** Mehr Jain, Nilita Sood, Innie Chen, Julia Rodrigues, Dalia Karol, Jun Y Hu, Melanie Altas, Faisal Khosa

**Affiliations:** 1 Department of Obstetrics and Gynaecology, The Ottawa Hospital, Ottawa, CAN; 2 Department of Medicine, The Ottawa Hospital, Ottawa, CAN; 3 Department of Family Medicine, McGill University, Montreal, CAN; 4 Department of Obstetrics and Gynaecology, University of Toronto, Toronto, CAN; 5 Department of Arts and Science, McGill University, Montreal, CAN; 6 Department of Obstetrics and Gynecology, University of British Columbia, Vancouver, CAN; 7 Department of Radiology, Vancouver General Hospital, Vancouver, CAN

**Keywords:** fellowship programs, program websites, rei, reproductive endocrinology and infertility, website comprehensiveness

## Abstract

The aim of the study was to assess the comprehensiveness of the Reproductive Endocrinology and Infertility (REI) fellowship program websites in North America. All active REI fellowship program websites in the United States of America (USA) and Canada were evaluated and assessed using 72-point scoring criteria. Any fellowship programs without publicly accessible websites were excluded. The scoring criteria consisted of the following domains - recruitment, faculty information, fellow information, research and education, surgical program, clinical work, benefits and career planning, wellness, and environment. We identified 49 REI fellowship programs in the USA and nine in Canada of which 47 A programs and all Canadian programs had an accessible website. The mean score was significantly higher for USA program websites (61.5% (USA) versus 47.7% (Canada); p<0.001). The "wellness" domain had the highest prevalence of criteria (85.3% of websites) across all program websites, whereas the "fellow information" domain had the lowest (20.0% of websites). In conclusion, American REI fellowship program websites included more program-related content, compared to Canadian program websites.

## Introduction

Medical trainees in all stages of their educational and professional careers rely on the information available on residency and fellowship program websites to help them assess and choose a program. A 2005 study with prospective emergency medicine residents showed that information on program websites influenced residents’ decisions to apply to specific programs [1]. However, recent studies about the content and comprehensiveness of various residency and fellowship program websites have documented room for improvement [2-9].

The Reproductive Endocrinology and Infertility (REI) fellowship program is a two to three-year sub-specialization training program, accredited by the Accreditation Council for Graduate Medical Education, which provides training to Obstetrics and Gynaecology (OBGYN) physicians in gynecological endocrinology, infertility and advanced reproductive technology, and reproductive surgery. There is a growing number of OBGYN residents choosing to sub-specialize, with about 10% of graduating OBGYN residents from the United States of America (USA) alone seeking fellowship training [10]. Accessibility and comprehensiveness of information on fellowship program websites have been vital for applicants, particularly in the time of the COVID-19 pandemic with the limitation/cancellation of visiting electives and travel restrictions due to public health guidelines. However, few studies have assessed the comprehensiveness of OBGYN fellowship program websites. In 2019, Sardana and co-workers evaluated information on Maternal Fetal Medicine (MFM) fellowship program websites and emphasized the need to improve the online presence of MFM fellowship programs [11]. Recently, our group found that, although urogynaecology fellowship program websites include some content relevant to applicants, there is room for improvement specifically information on clinical work and career planning [12].

Peyser et al. showed REI program websites to lack information [13]. To provide further insights into the content on program websites in order to identify specific information that websites should consider including given the demand and competitiveness of this subspecialty [14], this study assessed the comprehensiveness of North American REI fellowship program websites.

## Materials and methods

The study methodology has been validated in recent publications [5,6]. The study used publicly accessible data and did not involve human or animal subjects; therefore, Institutional Review Board approval was not required.

Data collection

A comprehensive list of USA and Canadian REI fellowship programs was compiled through the American Medical Association Fellowship and Residency Electronic Interactive Database (FRIEDA) and the Society of Obstetricians and Gynaecologists of Canada (SOGC) websites, respectively, in June 2020.

Website evaluation

The website scoring criteria comprised 72 items in the following domains - recruitment, faculty information, fellow information, research and education, surgical program, clinical work, benefits and career planning, wellness, and environment. One point was allocated if the corresponding information was available on the official program website, websites hyperlinked on the official page, or additional documents linked on the official program webpage. Websites published in French were translated through Google Translate prior to assessment against the scoring criteria.

Program websites were grouped based on geographic location. American program websites were subdivided into four regions - Northeast, Midwest, West, and South (Table 1). The Canadian fellowship programs were grouped together in the geographical analysis due to the limited number of programs in Canada. Census data were used to determine the number of programs per capita in the USA and Canada [15,16].

**Table 1:** US geographic locations categorized by region.

Northeast	Midwest	West	South
Connecticut	Illinois	Alaska	Alabama
Delaware	Indiana	Arizona	Arkansas
District of Columbia	Iowa	California	Florida
Maine	Kansas	Colorado	Georgia
Maryland	Michigan	Hawaii	Kentucky
Massachusetts	Minnesota	Idaho	Louisiana
New Hampshire	Missouri	Montana	Mississippi
New Jersey	Nebraska	Nevada	North Carolina
New York	North Dakota	New Mexico	Oklahoma
Pennsylvania	Ohio	Oregon	South Carolina
Rhode Island	South Dakota	Utah	Tennessee
Vermont	Wisconsin	Washington	Texas
-	-	Wyoming	Virginia

Statistical analysis

Descriptive statistics, including mean and standard deviation, were used to summarize website content comprehensiveness. REI fellowship program websites were classified by geographical location, and mean scores were compared using unpaired t-tests or ANOVA with Bonferroni correction for multiple comparisons. Statistical analysis was performed using Statistical Product and Service Solutions (SPSS, version 26; IBM SPSS Statistics for Windows, Armonk, NY).

## Results

A total of 57 REI fellowship programs were identified across North America (49 in the USA and nine in Canada). Of the 49 American programs, 47 programs had accessible websites, whereas all Canadian fellowship programs had accessible websites. Therefore, 55 programs were included in the website analysis.

Of the 49 REI fellowship programs in the USA, 20 (40.8%) were in the Northeast region of the USA, 11 (22.4%) in the Midwest, and nine (18.4%) each in the West and South. Northeast USA had the highest number of programs per capita (0.36 programs per million people), whereas West USA had the lowest (0.07 programs per million people) (Table 2).

**Table 2:** USA REI Fellowship Program information and website stats by geographical region. *n=18

Program Details/Website Criteria	Northeast USA	Midwest USA	West USA	South USA
Number of programs (n)	20	11	9	9
Number of programs (per million population)	0.36	0.16	0.07	0.12
Accreditation				
Accredited	20	11	9	9
Non-accredited	0	0	0	0
Website Criteria				
Overall criteria met	42.22 ± 7.74*	43.27 ± 6.68	48.11 ± 3.99	45.67 ± 7.00
%overall criteria met	58.6*	60.1	66.8	63.4
Recruitment criteria met	10.50 ± 2.18*	10.64 ± 2.16	12.44 ± 1.13	11.22 ± 2.33
Faculty Information criteria met	6.17 ± 1.54*	6.36 ± 2.16	6.22 ± 0.44	6.11 ± 1.69
Fellow Information criteria met	1.89 ± 1.53*	2.09 ± 1.51	3.00 ± 1.87	2.78 ± 1.39
Research and Education criteria met	6.11 ± 2.32*	6.91 ± 1.97	7.00 ± 1.32	6.67 ± 2.24
Surgical Program criteria met	4.00 ± 1.33*	4.36 ± 0.92	3.67 ± 0.87	4.33 ± 1.50
Clinical Work criteria met	1.83 ± 1.25*	1.45 ± 1.21	2.33 ± 1.00	2.11 ± 0.93
Benefits and Career Planning criteria met	5.44 ± 2.06*	5.36 ± 1.91	5.78 ± 1.56	5.44 ± 2.24
Wellness criteria met	2.44 ± 0.78*	2.73 ± 0.47	2.56 ± 0.53	2.89 ± 0.33
Environment criteria met	3.83 ± 1.04*	3.36 ± 1.91	5.11 ± 1.17	4.11 ± 1.05

The average website score among all REI program websites was 42.66±7.73 out of 72 items (59.3%). Mean website scores by geographical region are summarized in Table 3.

**Table 3:** Criteria Examined on USA and Canadian websites of Reproductive Endocrinology and Fertility Fellowship program websites. IMG - International Medical Graduate; OBGYN - Obstetrics and Gynecology; PGY - Postgraduate Year

Website Criteria	Websites Providing Information (n = 56)	Percentage of Websites Providing Information
Recruitment		
Contact email address for program	55	98.2
Mailing address	54	96.4
Selection criteria	47	83.9
Interview process	30	53.6
Interview dates	31	55.4
Electronic Application Service	47	83.9
Research requirements	0	0
Recruitment details	1	1.8
IMG information	34	60.7
Program description	56	100
Program director name	54	96.4
New fellows per year	19	33.9
Total OBGYN residents	49	87.5
Total fellows in subspecialty	37	66.1
Total staff/attending	54	96.4
Message from program director	9	16.1
Message from department chair	27	48.2
Faculty Information		
Comprehensive faculty listing	55	98.2
Specialty	54	96.4
Photos	53	94.6
Educational background	53	94.6
Research interests	52	92.9
Research publications	43	76.8
Awards	32	57.1
Research presentations	6	10.7
Fellow Information		
List of current fellows with names	39	69.6
Fellow year status (ex. PGY)	29	51.8
Individual or group photo	30	53.6
Past Alumni names	13	23.2
Alumni locations	7	12.5
Research and Education		
Research opportunities	56	100
Current research projects	23	41.1
Past research projects	19	33.9
Grants awarded	31	55.4
Journal club	33	58.9
Meeting/conference opportunities	44	78.6
Teaching	38	67.9
Grand round conferences	32	57.1
Educational resources	37	66.1
Research requirements	47	83.9
Surgical Program		
Responsibility progression	32	57.1
Surgical case	53	94.6
Surgical statistics	23	41.1
Imaging case numbers	2	3.6
Imaging equipment description	15	26.8
Ultrasound component	39	69.6
Simulation	18	32.1
Robotics	20	35.7
International opportunities	13	23.2
Clinical Work		
Expected case load	9	16.1
Rotation schedule	40	71.4
Work hours	16	28.6
Call Schedule	9	16.1
On call responsibilities	20	35.7
Evaluation	9	16.1
Benefits and Career Planning		
Incentives	44	78.6
Salary	28	50
Vacation	46	82.1
Maternal leave mentioned	38	679
Paternal leave mentioned	38	67.9
Moonlighting mentioned	29	51.8
Career placement	16	28.6
Future study opportunities	37	66.1
Wellness		
Fellow wellness	46	82.1
Professional organizations	56	100
Harassment Policy	44	48.6
Environment		
Hospitals	55	98.2
Neighborhood information	49	87.5
Local attractions	46	82.1
Social events	16	28.6
Pictures of social events	13	23.2
House options	40	71.4

USA fellowship program websites

The overall mean percent score of USA fellowship program websites was 61.47% (n=44.26±6.95 out of 72 criteria), with "wellness" as the highest scoring domain (87.33%, n=2.62±0.610 out of three criteria). Within this domain, information/mention about professional organizations was present across all 47 USA fellowship program websites. On the other hand, "clinical work" was the lowest scoring domain (31.50%, n=1.89±1.169 out of six criteria), with "expected case load," and "evaluation details" (15%, n=7/47) as the least reported criteria.

Information about "program description," "research opportunities," and "associated professional organizations" was present across all USA fellowship program websites. Beyond these, the most frequently included criteria were "program contact email address," "comprehensive faculty listing," "information on procedural case opportunities," and "affiliated hospital information" (98%, n=46/47). The last reported criteria were "recruitment details" (2%, n=1/47), "opportunities to practice imaging modalities and interpretation" (2%, n=1/47), "research presentations by faculty" (13%, n=6/47), and "current positions of alumni" (13%, n=6/47). Information about "research requirements for application" was not found on any USA fellowship program websites.

Canadian fellowship program websites

The overall mean percent score of Canadian fellowship program websites was 47.68% (n=34.33±6.33 out of 72 criteria), with "wellness" as the highest scoring domain (85.33%, n=2.56±0.726 out of three three criteria). Similar to USA program fellowship websites, information/mention about "associated professional organizations" was available on all Canadian fellowship program websites. "Fellow information" was the lowest scoring domain (20.0%, n=1±1.732 out of five criteria, with "past alumni names," and "current positions of alumni" as the least reported criteria (11%, n=1/9).

Information about "program contact information (email/mail address)," "selection criteria," "program description," "program director," "comprehensive faculty listing" (number of physicians with a subspecialty, photo, educational background, research interests), "research opportunities," "associated professional organizations," and "hospitals" was present across all Canadian fellowship program websites. The following criteria were not mentioned on any Canadian fellowship program website - "research requirements for application," "recruitment details," "message from the program director," "research presentations by faculty," "details regarding the imaging equipment available," "call schedule," and "career placement after completing program."

**Figure 1:**
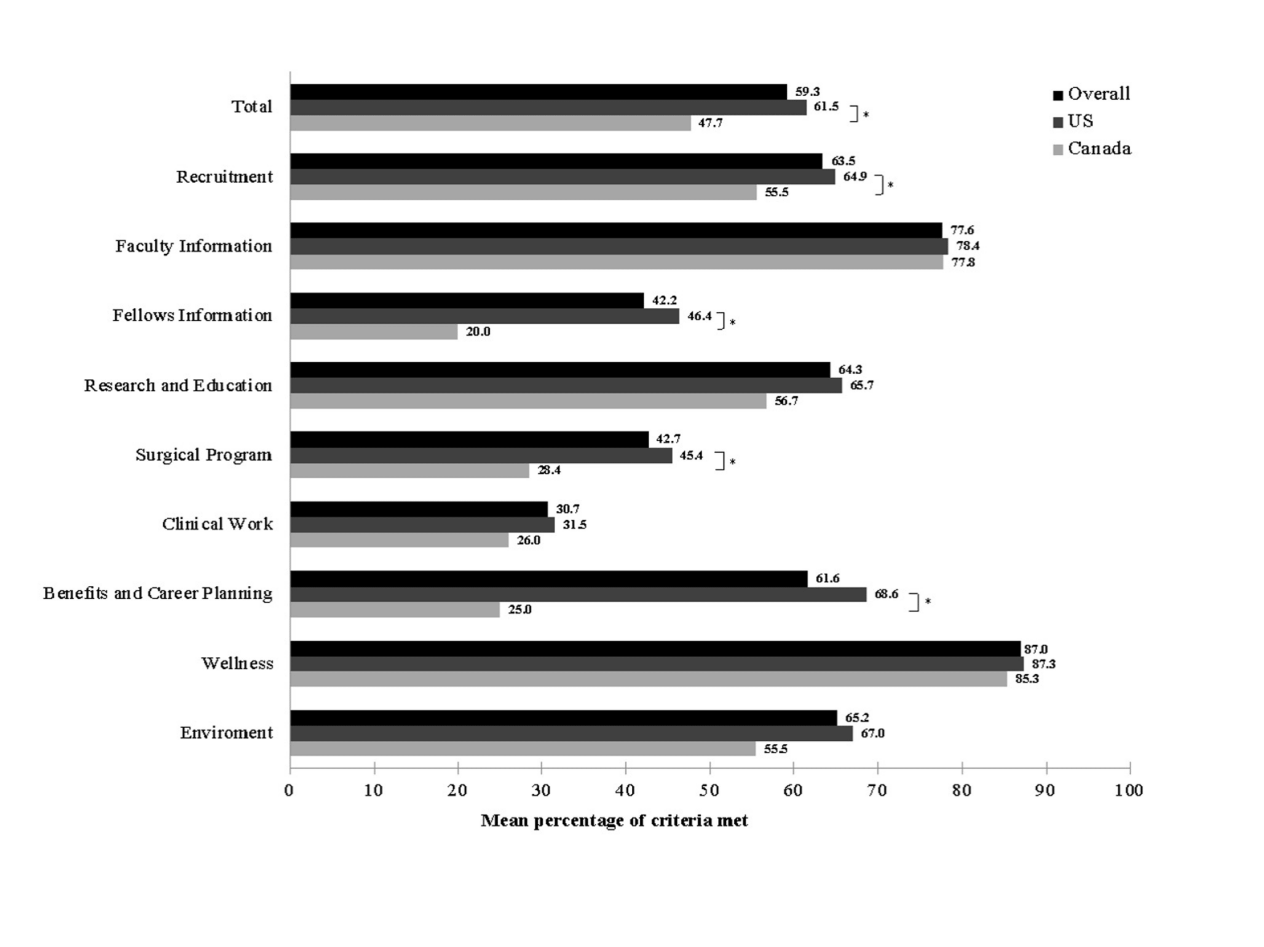
Mean percentage of criteria met for overall, USA, and Canadian REI fellowship program websites. *indicates significant differences.

Overall, Canadian fellowship programs had significantly lower website scores, compared to USA fellowship programs (Figure 1). USA program websites obtained significantly higher scores in the following domains - "recruitment," "fellow information," "surgical program," and "benefits and career planning" (Figure 1). Canadian fellowship program websites scored higher in the "faculty information" and "wellness" domains; however, differences were not statistically significant (Figure 1).

## Discussion

Since the onset of the COVID-19 pandemic, more applicants have turned to program websites for information about residency and fellowship programs due to limitations or hesitations in traveling. Program websites are a key source of information for applicants and a valuable opportunity for programs to attract prospective applicants. This study evaluated North American REI fellowship program websites to assess the comprehensiveness of the online information and present recommendations to improve online program representation. Overall, the "wellness" domain had the highest criteria prevalence (85.3%) across all program websites, whereas the "fellow information" domain had the lowest (20.0%). All program websites included details of "program description," "research opportunities," and association with "professional organizations." All but one website assessed included an email address to connect with the fellowship program.

Recently, Sardana et al.found that MFM fellowship program websites lacked important details relevant to applicants, which can often lead to increased emotional, financial, and administrative pressure [11]. Moreover, recent studies of North American Urogynecology program websites, and Canadian OB/GYN residency and fellowship program websites, note an overall lack of information that is needed for applicants to make informed training decisions [5,6]. Similar results were noted in recent studies of other medical subspecialties [2-4,7-10].

The highest-scoring "wellness" domain includes three criteria - "resident wellness," "associations with professional organizations, such as OMA and CMA," and "harassment policy." This result may be partially explained by the increased awareness of wellness and burnout prevention in medicine over the last several years. High stress and burnout are common among medical students, residents, and fellows, particularly during training years [17-20]. REI fellowship programs have kept their websites up-to-date in response to the potential for stress and burnout during the fellowship. Training programs should continue to present wellness resources to their applicants and trainees on their websites.

The data demonstrate that USA fellowship program websites scored significantly higher than Canadian ones. The "clinical work" domain had the lowest score among US REI fellowship program websites, with "expected caseload" and "evaluation details" as the least reported criteria. Details of clinical work provide applicants with insights into their future responsibilities as a fellow, often impacting their decision to apply to a program.

Among Canadian program websites, the lowest-scoring domain was information on "current fellows." In contrast, an analysis of MFM program websites demonstrated that 80.5% of websites mentioned the names of past fellows, and 85.4% of websites had information regarding current alumni employment [16]. Providing information about current and past trainees on program websites has been established to be valuable to prospective applicants [3].

Of note, details about "research requirements" for applicants to REI fellowship programs were not mentioned on any program website. Providing applicants with information about the expectation for demonstrating research productivity in the application process is important for trainees in deciding on which programs to apply to. Our results of REI program websites match the trend presented in the MFM program website evaluation, where only 6.1% of all websites mentioned programs’ research requirements [16]. On the contrary, a 2014 study of otolaryngology fellowship program websites showed that 69% of the program websites provided information on research requirements [21].

A strength of this study is the inclusion of a unique geographical analysis, including a per capita analysis of program distribution. Moreover, the website scoring criteria against which all fellowship program websites were evaluated underwent a vigorous developmental process and has already been published [5,6].

However, this study also has limitations. Firstly, the information assessed was only from program websites, and this excluded any information on online forums, email chains, or via word of mouth. The reviewers explored multiple links and attachments on the websites to explore all rating criteria, and it is possible that some information may have been missed. The use of two reviewers was used as a protective factor to counter this limitation. Another limitation of this study is that not all website criteria assessed in the study are of equal importance to the applicant. Minimal literature is available examining what website content is most important to fellowship applicants.

## Conclusions

In conclusion, given the transition to virtual information sessions and interviews after the COVID pandemic, information on fellowship program websites is vital for applicants. Although more than half of North American REI fellowship program websites thoroughly provide details about "wellness" and "faculty information," REI program websites lack other information pertinent to prospective applicants. Program websites did not include information on research requirements. American websites did not commonly include information on clinical work, while Canadian websites commonly did not include information on current fellows. Current REI websites can be further enhanced to include this information, to ensure a competitive advantage in recruiting the ideal candidates for their program, and to improve the applicant experience. Further studies should be conducted to explore the content REI fellowship applicants value in a program website.
